# Supplementary material for: Associations between air pollutants and blood pressure in an ethnically diverse cohort of adolescents in London, England
Source: PLoS One. 2023 Feb 8;18(2):e0279719. doi: 10.1371/journal.pone.0279719 (PMC9907839; doi:10.1371/journal.pone.0279719)
Supplement: S1 Fig — The ten boroughs in which the study took place are highlighted together with the locations of the participating schools (open triangles). (DOCX) [file pone.0279719.s001.docx]

**
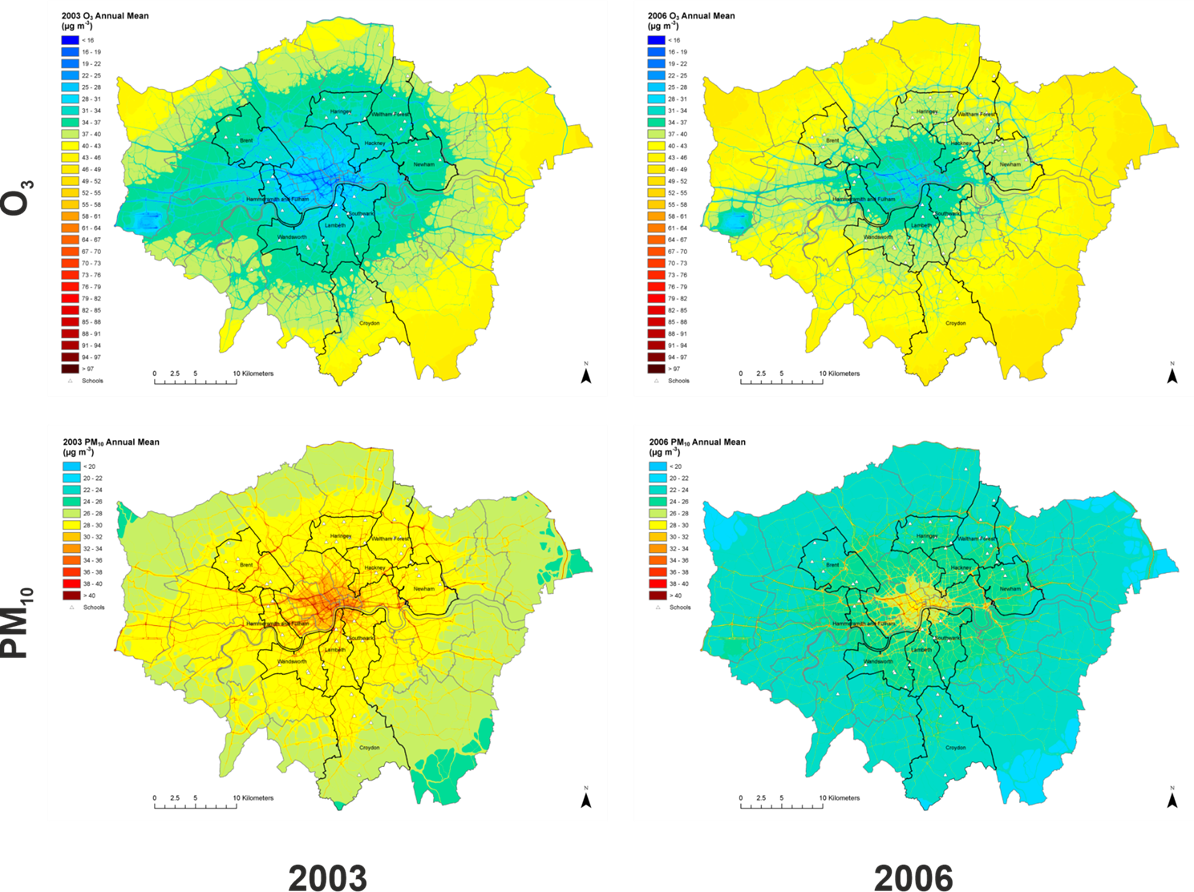
**

**S1 Figure**: Modelled Greater London concentrations (20m^2^ resolution) for O_3_ and PM_10_ for representative years 2003 and 2006. The ten boroughs in which the study took place are highlighted together with the locations of the participating schools (open triangles).
