# Supplementary material for: Associations between air pollutants and blood pressure in an ethnically diverse cohort of adolescents in London, England
Source: PLoS One. 2023 Feb 8;18(2):e0279719. doi: 10.1371/journal.pone.0279719 (PMC9907839; doi:10.1371/journal.pone.0279719)
Supplement: S2 Fig — For NO2 the data from the following number of sites were averaged over the period: Inner London RS (n = 6), outer London RS (n = 7), inner London BG (n = 4), outer London BG (n = 9). For PM10 the equivalent site number were n = 7, 10, 3 and 5, and for O3: n = 3, 3, 4 and 5. Insufficient sites monitored PM2.5 at the beginning of the study period and therefore trends are not shown. In each panel the rate of change in pollutant concentration is illustrated (mean with 95% confidence interval), as μg/m3 per year’. (DOCX) [file pone.0279719.s002.docx]

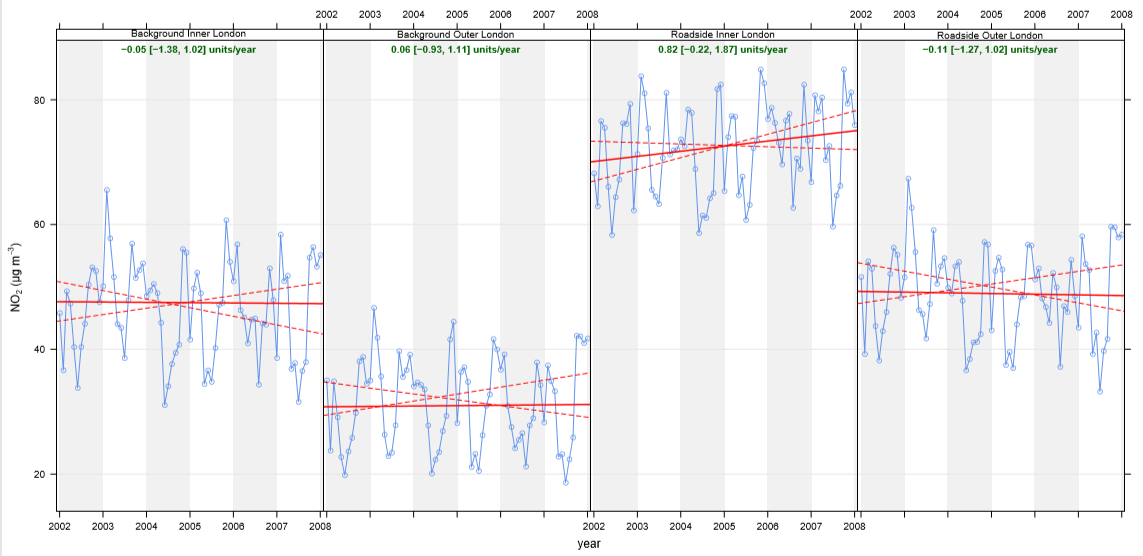

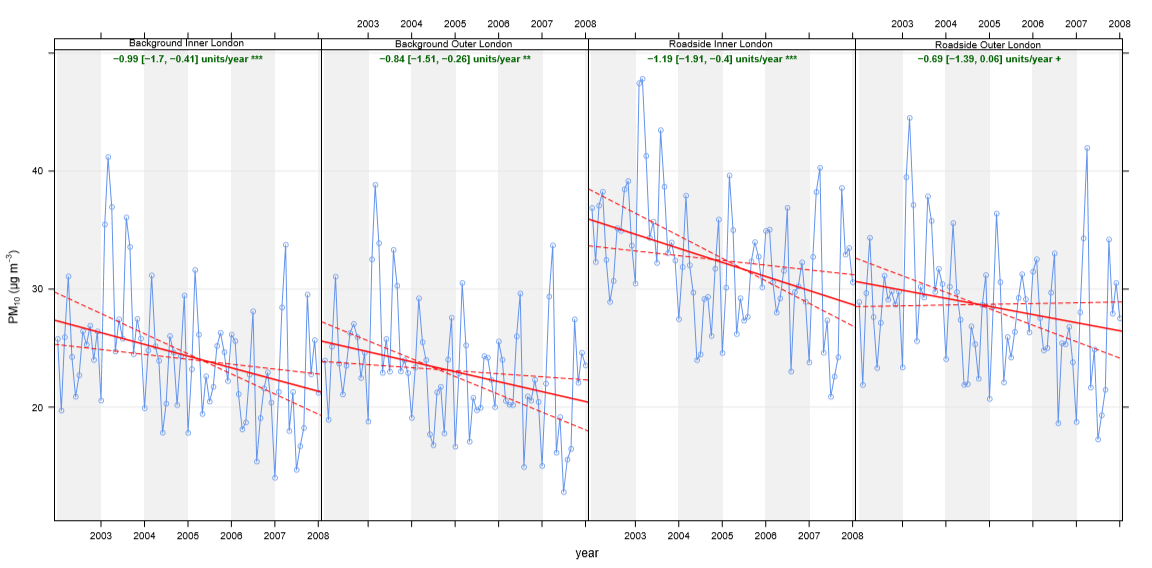

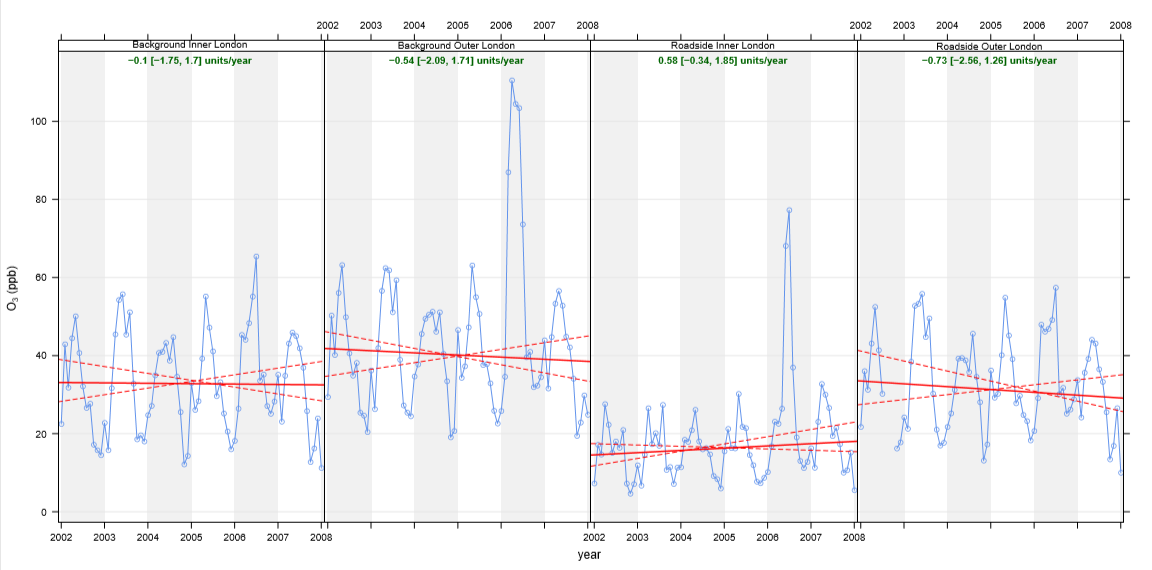


**S2 Figure:** Changes in monthly average concentrations of NO_2_, PM_10_ and O^3^ between 2002 and 2008 using sites classified as inner and outer London roadside (RS) and background (BG) locations. For NO_2_ the data from the following number of sites were averaged over the period: inner London RS (n=6), outer London RS (n=7), inner London BG (n=4), outer London BG (n=9). For PM_10_ the equivalent site number were n=7, 10, 3 and 5, and for O_3_: n=3, 3, 4 and 5. Insufficient sites monitored PM_2.5_ at the beginning of the study period and therefore trends are not shown. In each panel the rate of change in pollutant concentration is illustrated (mean with 95% confidence interval), as µg/m^3^ per year.’
