# Supplementary material for: Associations between air pollutants and blood pressure in an ethnically diverse cohort of adolescents in London, England
Source: PLoS One. 2023 Feb 8;18(2):e0279719. doi: 10.1371/journal.pone.0279719 (PMC9907839; doi:10.1371/journal.pone.0279719)
Supplement: S3 Fig — (DOCX) [file pone.0279719.s003.docx]

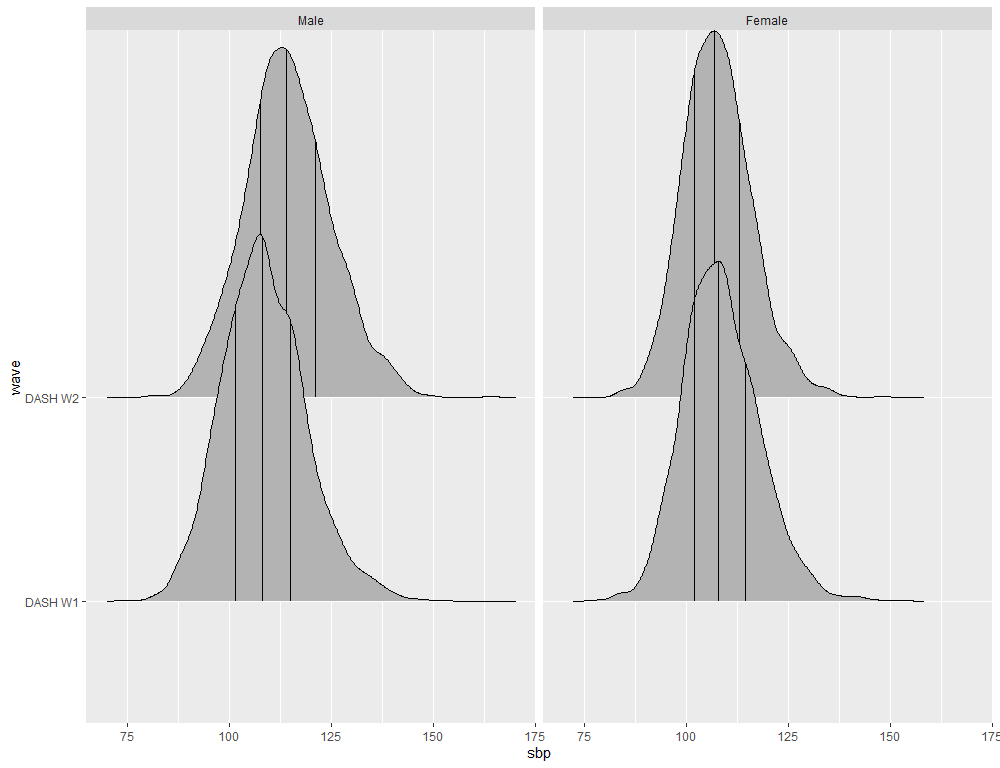


**S3 Figure:** Blood pressure distributions by sex and DASH sweep (vertical lines highlight the median and the interquartile range).
