# Supplementary figures and images for: Associations between air pollutants and blood pressure in an ethnically diverse cohort of adolescents in London, England
Source: PLoS One. 2023 Feb 8;18(2):e0279719. doi: 10.1371/journal.pone.0279719 (PMC9907839; doi:10.1371/journal.pone.0279719)

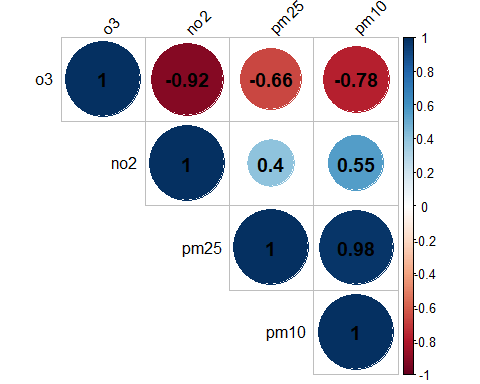


**S4 Figure** : Correlations between air pollutants

Supplement: S4 Fig — (DOCX) [file pone.0279719.s004.docx]
