# Supplementary material for: Associations between air pollutants and blood pressure in an ethnically diverse cohort of adolescents in London, England
Source: PLoS One. 2023 Feb 8;18(2):e0279719. doi: 10.1371/journal.pone.0279719 (PMC9907839; doi:10.1371/journal.pone.0279719)
Supplement: S1 Table — (DOCX) [file pone.0279719.s005.docx]

**S1 Table:** Differences between DASH participants with complete and missing information during the study period by selected variables

|  | Complete^1^ | Missing^1^ | p-value^2^ |
| --- | --- | --- | --- |
| **Systolic BP** | 108 (103, 116) | 110 (103, 116) | 0.011 |
| **Diastolic BP** | 68 (63, 72) | 68 (63, 72) | 0.6 |
| **PM_2.5_** (μg/m^3^) | 17.95 (15.90, 19.22) | 16.69 (15.91, 19.19) | 0.090 |
| **PM_10_** (μg/m^3^) | 27.19 (24.70, 28.59) | 26.20 (24.78, 28.54) | 0.13 |
| **NO_2_** (μg/m^3^) | 40.0 (38.3, 43.0) | 40.3 (38.6, 43.4) | <0.001 |
| **O_3_** (μg/m^3^) | 35.45 (33.67, 37.26) | 35.52 (33.56, 37.13) | 0.4 |
| **zBMI** | 0.37 (-0.46, 1.20) | 0.47 (-0.46, 1.44) | 0.013 |
| **zHeight** | 0.10 (-0.53, 0.77) | -0.01 (-0.69, 0.68) | <0.001 |
| **Sex** |  |  |  |
| Male | (47%) | (58%) | <0.001 |
| Female | (53%) | (42%) |  |
| **Ethnicity** |  |  |  |
| White | (34%) | (22%) | <0.001 |
| Black Caribbean | (19%) | (23%) |  |
| Black African | (21%) | (28%) |  |
| Indian | (13%) | (12%) |  |
| Pakistani/Bangladeshi | (12%) | (15%) |  |
| **Physical activity (number of activities)** | 8 (4, 19) | 9 (5, 20) | <0.001 |
| **Smoking** | (29%) | (30%) | 0.4 |
| **Alcohol** | (46%) | (38%) | <0.001 |
| **IMD score-Income domain^†^** | 0.24 (0.16, 0.34) | 0.28 (0.19, 0.36) | <0.001 |
| **Family Affluence Score** | 3.00 (2.00, 5.00) | 3.00 (2.00, 4.00) | <0.001 |
| **Family type** |  |  |  |
| 2 parent family, >=1 employed | (68%) | (62%) | <0.001 |
| 1 parent family, >=1 employed | (19%) | (18%) |  |
| 2 parent family, 0 employed | (6.0%) | (8.9%) |  |
| 1 parent family, 0 employed | (7.6%) | (10%) |  |

^1^Median (IQR); (%)

^2^Wilcoxon rank sum test; Pearson's Chi-squared test

^†^Index of Multiple Deprivation
