# Supplementary material for: Associations between air pollutants and blood pressure in an ethnically diverse cohort of adolescents in London, England
Source: PLoS One. 2023 Feb 8;18(2):e0279719. doi: 10.1371/journal.pone.0279719 (PMC9907839; doi:10.1371/journal.pone.0279719)
Supplement: S2 Table — (DOCX) [file pone.0279719.s006.docx]

**S2 Table:** Selected descriptive characteristics of the DASH participants (complete cases)

|  | **Boys 11-13 years** | **Girls 11-13 years** | **Boys 14-16 years** | **Girls 14-16 years** |
| --- | --- | --- | --- | --- |
|  | **(n=867)** | **(n=993)** | **(n=867)** | **(n=993)** |
| **Blood Pressure** |  |  |  |  |
| Mean systolic (95% CI) | 108 (107 to 109) | 108 (108 to 109) | 114 (113 to 115) | 107 (107 to 108) |
| % > or = 90^th^ Centile^§^ (95% CI) | 2.8 (1.7 to 4.4) | 1.3 (0.7 to 2.6) | 6.8 (5.0 to 9.1) | 1.2 (0.6 to 2.4) |
| Mean diastolic (95% CI) | 65 (65 to 66) | 68 (68 to 69) | 68 (68 to 69) | 68 (68 to 69) |
| % > or = 90^th^ Centile^§^(95% CI) | 1 (0.4 to 2.3) | 1.4 (0.7 to 2.6) | 1.4 (0.7 to 2.8) | 1.6 (0.9 to 3) |
| **Elevated systolic/diastolic Blood pressure** |  |  |  |  |
| % > or = 90^th^ Centile^§^ (95% CI) | 3.5 (2.2 to 5.3) | 2.6 (1.6 to 4.1) | 7.3 (5.4 to 9.3) | 2.1 (1.2 to 3.6) |
| **Air Pollutants** |  |  |  |  |
|  | **Median (IQR)** | **Median (IQR)** | **Median (IQR)** | **Median (IQR)** |
| NO_2_(μg/m^3^) | 40.4 (38.9 to 43.1) | 40.7 (38.9 to 43.3) | 39.7 (38.1 to 42.9) | 39.3 (37.5 to 42.8) |
| PM _2.5_ (μg/m^3^) | 19.2 (19 to 19.7) | 19.2 (19 to 19.7) | 15.9 (15.6 to 16.3) | 15.9 (15.7 to 16.4) |
| PM_10_ (μg/m^3^) | 28.6 (28.1 to 29.3) | 28.6 (28.2 to 29.4) | 24.8 (24.3 to 25.5) | 24.6 (24.2 to 25.5) |
| O_3_ (μg/m^3^) | 34.6 (32.7 to 35.6) | 34.4 (32.7 to 35.7) | 36.7 (34.8 to 37.9) | 37.1 (35.1 to 38.9) |
|  | **Mean % (95% CI)** | | | |
| zHeight | 0 (-0.03 to 0.12) | 0.1 (0.05 to 0.2) | 0.19 (0.12 to 0.27) | 0.1 (0.01 to 0.18) |
| **zBMI** | 0.5 (0.34 to 0.56) | 0.61 (-0.14 to 1.4) | 0.15 (0.04 to 0.26) | 0.25 (0.23 to 0.42) |
| **Room temperature** | 23 (21 to 25) | 23 (22 to 24) | 21 (21 to 21) | 21 (19 to 23) |
| **Family Affluence Score^+^** |  |  |  |  |
| Least disadvantaged (Highest tertile) | 26 (22.6 to 29.8) | 20.7 (17.8 to 24) | 29 (25.4 to 32.8) | 27.3 (24 to 30.8) |
| Most disadvantaged (Lowest tertile) | 51.7 (47.6 to 55.8) | 35.7 (32.1 to 39.4) | 48.3 (44.2 to 52.4) | 54.0 (50.1 to 57.8) |
| **Family type+** |  |  |  |  |
| 2-parent family, >=1 employed | 72.3 (68 to 76) | 64.7 (61 to 68) | 70.5 (67 to 74) | 65 (61 to 69) |
| lone-parent family, 0 employed | 9.8 (7.5 to 13) | 10.5 (8.3 to 13) | 5.2 (3.6 to 7.5) | 4.8 (3.3 to 6.8) |
| **Physical activity (number of activities) ^+^** |  |  |  |  |
| Highest quartile | 28.1 (24.6 to 31.2) | 37 (33.4 to 40.8) | 26.9 (23.4 to 30.7) | 27.7 (24.4 to 31.3) |
| Lowest quartile | 16.5 (13.7 to 19.8) | 20.7 (17.8 to 24.0) | 23.4 (20.1 to 27) | 24.5 (21.4 to 28.0) |
| **Alcohol intake (%Yes)** | 33.7 (30 to 38) | 39.4 (36 to 43) | 48 (44 to 52) | 62.9 (59 to 67) |
| **Tobacco smoking (%Yes)** | 20.2 (17 to 24) | 17.9 (15 to 21) | 35.3 (31 to 39) | 42.5 (38 to 46) |
| **Pubertal stage (Late)** | 46.6 (42 to 51) | 54.1 (50 to 58) | 90.1 (87 to 92) | 89.8 (87 to 92) |
| **IMD-Income domain)^†+^** |  |  |  |  |
| Least deprived quartile | 26.2 (22.8 to 30) | 24.8 (21.7 to 28.0) | 26.9 (23.4 to 30.7) | 25.5 (22.3 to 28.9) |
| Most deprived quartile | 23.8 (20.5 to 27.7) | 27.0 (23.7 to 30.5) | 24.4 (21.1 to 28.2) | 24.8 (21.7 to 28.3) |
