# Supplementary material for: Associations between air pollutants and blood pressure in an ethnically diverse cohort of adolescents in London, England
Source: PLoS One. 2023 Feb 8;18(2):e0279719. doi: 10.1371/journal.pone.0279719 (PMC9907839; doi:10.1371/journal.pone.0279719)
Supplement: S3 Table — (DOCX) [file pone.0279719.s007.docx]

**S3 Table** Additional descriptive characteristics of the sample (multiply imputed)

|  | **Boys 11-13 years** | **Girls 11-13 years** | **Boys 14-16 years** | **Girls 14-16 years** |
| --- | --- | --- | --- | --- |
|  | **(n=1774)** | **(n=1549)** | **(n=1774)** | **(n=1549)** |
|  | **Mean (95% CI)** | **Mean (95% CI)** | **Mean (95% CI)** | **Mean (95% CI)** |
| **Mean BMI** | 19.9 (19.7 to 20.1) | 21.0 (20.9 to 21.2) | 20.9 (20.6 to 21.0) | 22.0 (21.7 to 22.2) |
| **zBMI** | 0.5 (0.4 to 0.6) | 0.6 (0.6 to 0.7) | 0.2 (0.1 to 0.3) | 0.4 (0.3 to 0.5) |
| **Mean Height** | 155.2 (154.7 to 155.6) | 156.3 (155.9 to 156.7) | 170.9 (170.5 to 171.2) | 162.3 (162.0 to 162.7) |
| **zHeight** | 0.0 (-0.1 to 0.0) | 0.0 (0.0 to 0.1) | 0.1 (0.1 to 0.2) | 0.0 (0.0 to 0.1) |
| **Ambient air temperature^§^** | 22.4 (22.2 to 22.6) | 22.4 (22.2 to 22.5) | 20.5 (20.3 to 20.6) | 20.5 (20.3 to 20.6) |
| Lung function at 11-13y |  |  |  |  |
| FEV_1_, L | 2.40 (2.37 to 2.43) | 2.38 (2.35 to 2.40) | - | - |
| FVC, L | 2.89 (2.85 to 2.93) | 2.78 (2.75 to 2.81) | - | - |
| Noise pollution |  |  |  |  |
| Lden (dB) | 60.3 (60.1 to 60.6) | 60.4 (60.2 to 60.7) | 60.4 (60.1 to 60.7) | 60.5 (60.3 to 60.8) |
| **Family Affluence** |  |  |  |  |
| Least disadvantaged (Highest tertile) | 22.2 (20.1 to 24.3) | 17.5 (15.5 to 19.5) | 26.4 (24.3 to 28.6) | 23.5 (21.3 to 25.8) |
| Less disadvantaged (Medium tertile) | 19.7 (17.6 to 21.7) | 42.0 (39.4 to 44.6) | 20.7 (18.7 to 22.6) | 43.6 (41.0 to 46.1) |
| Most disadvantaged (Lowest tertile) | 58.1 (55.6 to 60.6) | 40.5 (37.8 to 43.1) | 52.9 (50.5 to 55.4 | 32.9 (30.4 to 35.6) |
| **Family type** |  |  |  |  |
| 2-parent family, >=1 employed | 66.3 (66.0 to 66.6) | 61.3 (61.0 to 61.6) | 66.1 (65.9 to 66.4) | 61.1 (60.8 to 61.4) |
| 2-parent family, 0 employed | 8.4 (8.2 to 8.6) | 7.5 (7.3 to 7.7) | 8.2 (8.0 to 8.3) | 7.5 (7.3 to7.6) |
| 1-parent family, >=1 employed | 14.4 (14.2 to 14.7) | 18.7 (18.5 to 19.0) | 18.4 (18.4 to 18.8) | 24.0 (23.7 to 24.3) |
| 1-parent family, 0 employed | 10.8 (10.7 to 11.0) | 12.5 (12.3 to 12.7) | 7.1 (6.9 to 7.2) | 7.4 (7.1 to 7.6) |
| **Physical activity (number of activities)** |  |  |  |  |
| Lowest quartile | 15.9 (14.1 to 17.7) | 35.6 (33.1 to 38.2) | 23.3 (21.3 to 25.4) | 32.3 (29.8 to 34.7) |
| 2^nd^ | 27.7 (25.4 to 30.0) | 27.7 (25.3 to 30.1) | 21.1 (19.1 to 23.0) | 24.3 (22.1, 26.5) |
| 3^rd^ | 25.4 (23.2 to 27.6) | 21.5 (19.3 to 23.7) | 27.1 (25.0 to 29.2) | 24.1 (21.9, 26.3) |
| Highest quartile | 31.0 (29.6 to 33.4) | 15.1 (13.2 to 17.0) | 28.5 (26.4 to 30.7) | 19.3 (17.3, 21.4) |
| **IMD-Income domain^†^** |  |  |  |  |
| Least deprived quartile | 31.5 (29.3 to 33.7) | 30.2 (27.8 to 32.5) | 22.4 (20.4 to 24.4) | 22.1 (19.9 to 24.2) |
| 2^nd^ | 26.5 (24.4 to 28.6) | 23.9 (21.8 to 26.1) | 27.7 (25.5 to 29.0) | 24.6 (22.4 to 26.9) |
| 3^rd^ | 23.1 (21.1 to 25.1) | 23.1 (20.9 to 25.3) | 22.3 (20.3 to 24.3) | 22.8 (20.6 to 25.0) |
| Most deprived quartile | 18.9 (17.0 to 20.8) | 22.8 (20.6, 24.9) | 27.6 (25.5 to 29.8) | 30.5 (28.1 to 32.9) |

^§^ Ambient air temperature in Celsius degrees

^†^Index of Multiple Deprivation
