# Supplementary material for: Associations between air pollutants and blood pressure in an ethnically diverse cohort of adolescents in London, England
Source: PLoS One. 2023 Feb 8;18(2):e0279719. doi: 10.1371/journal.pone.0279719 (PMC9907839; doi:10.1371/journal.pone.0279719)
Supplement: S4 Table — (DOCX) [file pone.0279719.s008.docx]

**S4 Table:** Longitudinal associations between air pollutants and Blood Pressure among participants aged ll-16 years in the DASH study (Complete Cases)

|  |  | **Boys** | | | | **Girls** | | | |
| --- | --- | --- | --- | --- | --- | --- | --- | --- | --- |
|  |  | **Single-Pollutant** | | **Two-pollutant** | | **Single-Pollutant** | | **Two-pollutant** | |
| **Pollutants** |  |  |  |  |  |  |  |  |  |
| **Systolic BP** | | **β (95% CI)** | **P>\|z\|** | **β (95% CI)** | **P>\|z\|** | **β (95% CI)** | **P>\|z\|** | **β (95% CI)** | **P>\|z\|** |
| **O_3_** (μg/m^3^) | Model 1 | 0.13 (-0.01 to 0.28) | 0.084 | - | - | 0.18 (0.04 to 0.32) | 0.013 | - | - |
|  | Model 2 | 0.18 (0.02 to 0.34) | 0.025 | - | - | 0.16 (0.01 to 0.32) | 0.033 | - | - |
| **NO_2_** (μg/m^3^) | Model 1 | -0.08 (-0.18 to 0.01) | 0.089 | -0.18 (-0.33 to 0.04) | 0.013 | -0.16 (-0.26 to -0.07) | <0.001 | -0.29 (-0.40 to -0.18) | <0.001 |
|  | Model 2 | -0.11 (-0.20 to -0.01) | 0.032 | -0.21 (-0.35 to -0.09) | 0.002 | -0.16 (-0.26 to -0.06) | 0.001 | -0.32 (-0.45 to -0.20) | <0.001 |
| **PM _2.5_** (μg/m^3^) | Model 1 | 0.11 (-0.32 to 0.54) | 0.617 | 0.58 (-0.08 to 1.24) | 0.086 | 0.30 (-0.16 to 0.76) | 0.198 | 1.36 (0.84 to 1.88) | <0.001 |
|  | Model 2 | 0.05 (-0.38 to 0.49) | 0.817 | 0.58 (-0.03 to 1.20) | 0.063 | 0.39 (-0.07 to 0.86) | 0.102 | 1.31 (0.73 to 1.88) | <0.001 |
| **PM _10_** (μg/m^3^) | Model 1 | -0.05 (-0.35 to 0.24) | 0.718 | - | - | -0.04 (-0.34 to 0.26) | 0.805 | - | - |
|  | Model 2 | -0.10 (-0.40 to 0.19) | 0.493 | - | - | 0.02 (-0.29 to 0.33) | 0.894 | - | - |
| **Diastolic BP** | | **β (95% CI)** | **P>\|z\|** | **β (95% CI)** | **P>\|z\|** | **β (95% CI)** | **P>\|z\|** | **β (95% CI)** | **P>\|z\|** |
| **O_3_** (μg/m^3^) | Model 1 | 0.00 (-0.11 to 0.11) | 0.961 | - | - | 0.10 (-0.01 to 0.20) | 0.074 | - | - |
|  | Model 2 | -0.02 (-0.13 to 0.10) | 0.732 | - | - | 0.07 (-0.04 to 0.19) | 0.231 | - | - |
| **NO_2_** (μg/m^3^) | Model 1 | 0.00 (-0.07 to 0.07) | 0.928 | 0.01 (-0.10 to 0.12) | 0.888 | -0.09 (-0.16 to -0.02) | 0.01 | -0.07 (-0.17 to 0.02) | 0.131 |
|  | Model 2 | 0.01 (-0.06 to 0.09) | 0.69 | -0.03 (-0.13 to 0.07) | 0.603 | -0.07 (-0.14 to 0.00) | 0.062 | -0.07 (-0.16 to 0.02) | 0.14 |
| **PM _2.5_** (μg/m^3^) | Model 1 | 0.04 (-0.28 to 0.36) | 0.796 | 0.07 (-0.42 to 0.57) | 0.774 | -0.30 (-0.66 to 0.05) | 0.093 | -0.01 (-0.49 to 0.46) | 0.956 |
|  | Model 2 | 0.09 (-0.23 to 0.42) | 0.588 | 0.11 (-0.35 to 0.57) | 0.64 | -0.21 (-0.56 to 0.15) | 0.253 | -0.01 (-0.46 to 0.43) | 0.957 |
| **PM _10_** (μg/m^3^) | Model 1 | 0.03 (-0.18 to 0.25) | 0.764 | - | - | -0.24 (-0.48 to -0.01) | 0.04 | - | - |
|  | Model 2 | 0.06 (-0.16 to 0.28) | 0.595 | - | - | -0.19 (-0.43 to 0.05) | 0.122 | - | - |

Model 1: coefficients were estimated with random effects models, adjusted for age, age^2^ zBMI, zHeight, ambient air temperature and pubertal stage.

Model 2: model1+ ethnicity, alcohol, smoking, physical activity, family type and parental employment, family affluence score and neighbourhood deprivation
