# Supplementary material for: Associations between air pollutants and blood pressure in an ethnically diverse cohort of adolescents in London, England
Source: PLoS One. 2023 Feb 8;18(2):e0279719. doi: 10.1371/journal.pone.0279719 (PMC9907839; doi:10.1371/journal.pone.0279719)
Supplement: Attachment — Submitted filename: Response_plosone.docx [file pone.0279719.s009.docx]

We thank the editorial team as well as the reviewers of our manuscript for their comments. Below are our response to reviewers’ comments.

**Reviewer #1**

1. *Line 157-158 Page 6: The description is not clear that “all exposures used the annual mean within a 20m radius buffer zone”. Given the study conducted during 2003-2006, was the exposure the annual mean level of pollutants at the year of baseline survey, or the average during 2003-2006*

Response

Exposures were estimated for the year in which the assessment was performed.

2. *The exposure assignment was according to each child’s residential postcode. How about the proportion of the study participants to migrate other places during the follow-up?*

Response

Fewer than 1% of participants moved homes during the study period.

3. *In Supplemental Table 1, the comparison showed a higher proportion for boys in the missing group than the complete group. I did not know whether potential selection bias might affect the stronger pollution-BP associations observed in the study. Please add some discussions or limitations.*

Response

We have updated the text of our manuscript as requested Please see lines 392-396.

4. *The concentrations of air pollutants are related to local relative humidity beyond ambient temperature. In the statistical analysis, could the associations be further adjusted for relative humidity across study locations?*

We have employed humidity in London based studies previously. It has not made any difference to the results. I think there is no need to do this.

**Reviewer #2**

5. *Line 116 Please remove text, “and numbers”.*

*Response*

Addressed as requested.

6. *Table 2 & line 265: Is there any known reason of higher exposure O3 in UK and Indian participants? Are there any statistical relation it with BP, for the respective age window in which higher exposure to O3 is observed.*

Response

Simply because of the inverse relationship with NO2, but it also reflects the distribution of ethnic groups throughout London. There is a very large Indian community in outer West London.

7. *Line 304. “The” is subscript*

*Response*

Addressed as requested.

8. *Line 303: An adjustment?*

*Response*

*Replaced with the word “adjustment”*

9. *Although limitation section is well written, but authors must address that atopic and other diseases that can affect the cardiovascular profile in large population is not included in models used in this study.*

*Response*

Please see lines 392-397

**Notes for the editor:**

- We tried to make the figure 1 more legible by splitting it to manuscript’s figure 1 and supplementary figure 1. Unfortunately, the newly attached figures is the best we could do.
- We have addressed some minor aesthetic issues regarding capitalisation; however, we were unable to address the issue of superscripts as Stata does not accept superscripts when generating graphs.
- a)	All figures were created by IS M, FJ K, SD B and D D. Figures were produced from their own in-house model using ArcGIS software. Maps were acquired (freely available) from the London data store.
- b)	Map images have not been previously copyrighted.
